# Identification of intergenerational epigenetic inheritance by whole genome DNA methylation analysis in trios

**DOI:** 10.1038/s41598-023-48517-3

**Published:** 2023-12-02

**Authors:** Anna Díez-Villanueva, Berta Martín, Ferran Moratalla-Navarro, Francisco D. Morón-Duran, Iván Galván-Femenía, Mireia Obón-Santacana, Anna Carreras, Rafael de Cid, Miguel A. Peinado, Victor Moreno

**Affiliations:** 1 Unit of Biomarkers and Susceptibility (UBS), Oncology Data Analytics Program (ODAP), Catalan Institute of Oncology (ICO), L’Hospitalet del Llobregat, 08908 Barcelona, Spain; 2 ONCOBELL Program, Bellvitge Biomedical Research Institute (IDIBELL), L’Hospitalet de Llobregat, 08908 Barcelona, Spain; 3 Consortium for Biomedical Research in Epidemiology and Public Health (CIBERESP), 28029 Madrid, Spain; 4 Germans Trias i Pujol Institute (IGTP), Translational Program in Cancer Research (CARE), Camí de les Escoles, s/n, Can Ruti Biomedical Campus, 08916 Badalona, Catalonia Spain; 5 Department of Clinical Sciences, Faculty of Medicine and Universitat de Barcelona Institute of Complex Systems (UBICS), University of Barcelona, 08907 Barcelona, Spain; 6 Genomes for Life-GCAT lab., Germans Trias i Pujol Research Institute (IGTP), Camí de les Escoles, s/n, Can Ruti Biomedical Campus, 08916 Badalona, Catalonia Spain

**Keywords:** DNA methylation, Epigenetic memory

## Abstract

Genome-wide association studies have identified thousands of loci associated with common diseases and traits. However, a large fraction of heritability remains unexplained. Epigenetic modifications, such as the observed in DNA methylation have been proposed as a mechanism of intergenerational inheritance. To investigate the potential contribution of DNA methylation to the missing heritability, we analysed the methylomes of four healthy trios (two parents and one offspring) using whole genome bisulphite sequencing. Of the 1.5 million CpGs (19%) with over 20% variability between parents in at least one family and compatible with a Mendelian inheritance pattern, only 3488 CpGs (0.2%) lacked correlation with any SNP in the genome, marking them as potential sites for intergenerational epigenetic inheritance. These markers were distributed genome-wide, with some preference to be located in promoters. They displayed a bimodal distribution, being either fully methylated or unmethylated, and were often found at the boundaries of genomic regions with high/low GC content. This analysis provides a starting point for future investigations into the missing heritability of simple and complex traits.

## Introduction

Heritability measures how much of the differences in biological traits within a population are influenced by genetic variation among individuals. These differences can arise from both genetic and environmental factors, including epigenetic influences. While the majority of genetic factors are inherited in a Mendelian fashion from one generation to the next, the mechanisms by which non-genetic factors influencing phenotypic variation can be transmitted across generations is a topic of intense research^1^.

In the past two decades, sequencing thousands of genomes has allowed us to uncover the genetic determinants of many diseases, including both Mendelian disorders such as cystic fibrosis, sickle cell anaemia, and Huntington's disease and complex prevalent diseases influenced by multiple genes and environmental factors such as heart disease, diabetes, and many cancers^2^. Genome-wide association studies (GWAS) have unveiled thousands of SNPs that have been associated with common diseases and traits^3^. However, the contribution of these studies to explain the heritability of common diseases has been limited, leading the scientific community to consider other mechanisms for finding the missing heritability^4,5^. Rare variants, especially in regions of low linkage disequilibrium^6^, structural variants^7^ as well as gene–gene and gene-environment interactions^8,9^ theoretically may contribute significantly to the unexplained heritability in complex traits and diseases^10^. The reality is that the explained heritability still is limited, which can be attributed in part to the difficulty in assessing environmental factors without measurement error, and the need for very large sample sizes to better detect rare genetic variations and gene-environment interactions^11^.

The contribution of non-genetic factors to inheritance has not been quantified and still is an area of research of mechanisms^1^. It is known that environmental factors such as diet^12,13^, temperature^14^, and famine^15^, among others, can modulate the inherited phenotype without altering the genetic sequence of an individual. DNA methylation, a prominent epigenetic modification predominantly occurring in cytosines within CpG dinucleotides, is a potential mechanism of interest. This modification plays a crucial role in the regulation of gene expression and fundamental cellular processes^16^. Additionally, DNA methylation is implicated in genomic processes related to inheritance, including imprinting and X chromosome inactivation, and serves as a key regulator of cell differentiation^17^. It is known that the methylation of cytosines occurs in response to environmental factors and affects phenotypes through changes in gene expression^18^. Although gametogenesis involves epigenetic reprogramming, certain DNA alterations induced by environmental factors have the potential to be passed down to subsequent generations, leading to transgenerational inheritance of DNA methylation^19–21^. While this mechanism is well-established in plants^22^, evidence in mammals, and particularly in humans, is still accumulating^20,23^, and remains contentious^24,25^. This is largely because most human studies are observational, which could be influenced by a multitude of confounding factors, and observation in animal models may have alternative explanations.

Here we aim to explore the hypothesis that intergenerational inheritance of DNA methylation, a poorly studied phenomenon in humans, may be a potential source of the missing heritability. To that extent, as a proof-of-principle, we identify specific CpG sites that follow a Mendelian inheritance pattern but without an apparent association with SNPs, thus becoming candidates of epigenetic inheritance.

## Materials and methods

### Study participants

In this proof-of-principle study, twelve samples from four healthy trios (parents and one offspring) were analysed. Subjects were participants in the GCAT cohort^26^, a prospective study that recruited almost 20,000 participants from the general population of Catalonia. All participants were over 18 and signed an informed consent agreement form and answered a comprehensive epidemiological questionnaire. The study was approved by the Hospital de Bellvitge Ethics Committee and by the Germans Trias i Pujol University Hospital ethics committee (reference PR275/15). All the procedures were performed in accordance with relevant guidelines and regulations. Table in Additional file 1 shows the family, family member, sex, and age for each sample.

### DNA extraction

Twenty millilitres of whole blood from each patient were collected in tubes with anticoagulant and stabilising agent (EDTA). Erythrocytes were depleted with BD Pharm Lyse TM lysing buffer. Cells were stained with PE-CyTM Mouse Anti-Human CD16 (BD-REF 557744) to sort the neutrophils subpopulation. Cells were sorted by FACS and DNA extraction was performed using a Maxwell 16 blood DNA purification Kit (ref AS1020) (Qiagen, Valencia, CA). After extraction, total DNA was quantified using a Qubit dsDNAHS Assay kit (Life Technologies, Grand Island, NY, USA). All DNA samples were stored at − 80 °C before sequencing library construction.

### Whole-genome bisulphite sequencing

DNA methylation was analysed at single-base resolution using whole-genome bisulphite sequencing (WGBS), yielding methylation information of more than 28 million CpGs. WGBS is based on the fact that when treating DNA with bisulphite, unmethylated cytosines (C) are transformed to thymines (T) while methylated ones remain as cytosines^27^.

Briefly, genomic DNA (2 μg) was spiked with unmethylated bacteriophage λ DNA (5 ng of λ DNA per microgram of genomic DNA; Promega), the gDNA was shared by sonication using a LE220 Focused-ultrasonicator (Covaris) and the DNA libraries were constructed using the TruSeq Sample Preparation kit (Illumina) following Illumina's standard protocol. After adaptor ligation, DNA was treated with sodium bisulphite using the EpiTect Bisulphite Kit (Qiagen). Enrichment for adaptor-ligated DNA was carried out through seven PCR cycles using the PfuTurboCx Hotstart DNA polymerase (Stratagene). Library quality was monitored using the Agilent 2100 Bioanalyzer (Agilent Technologies) using the Agilent DNA 7500 kit (Agilent Technologies). The concentration was estimated by KAPA Library Quantification Kit Illumina® Platforms (Kapa Biosystems).

Paired-end DNA sequencing (2 × 101 bp) of the converted libraries was performed using the HiSeq2000 (Illumina) following the manufacturer’s protocol with HiSeq Control Software (HCS) 1.5.15.1. Images analysis, base calling and quality scoring of the run were processed using the manufacturer’s software Real Time Analysis (RTA 1.13.48) and followed by generation of FASTQ sequence files. We aimed for a minimum average coverage of 10 × per sample (Additional file 1).

### WGBS analysis pipeline and quality control

We used wg-blimp^28^, a pipeline to analyse WGBS data that integrates established algorithms for alignment, quality control and methylation calling among other functions. The software wg-blimp only requires a reference genome and fastq raw sequencing files as input. The percentage of methylation of 28,201,580 CpGs was obtained for each sample. Table in Additional file 1 shows the quality control metrics from Qualimap^29^, FastQC^30^ and Picard (https://broadinstitute.github.io/picard/) obtained from the wg-blimp alignment.

### CpGs filtering

**Figure 1:**
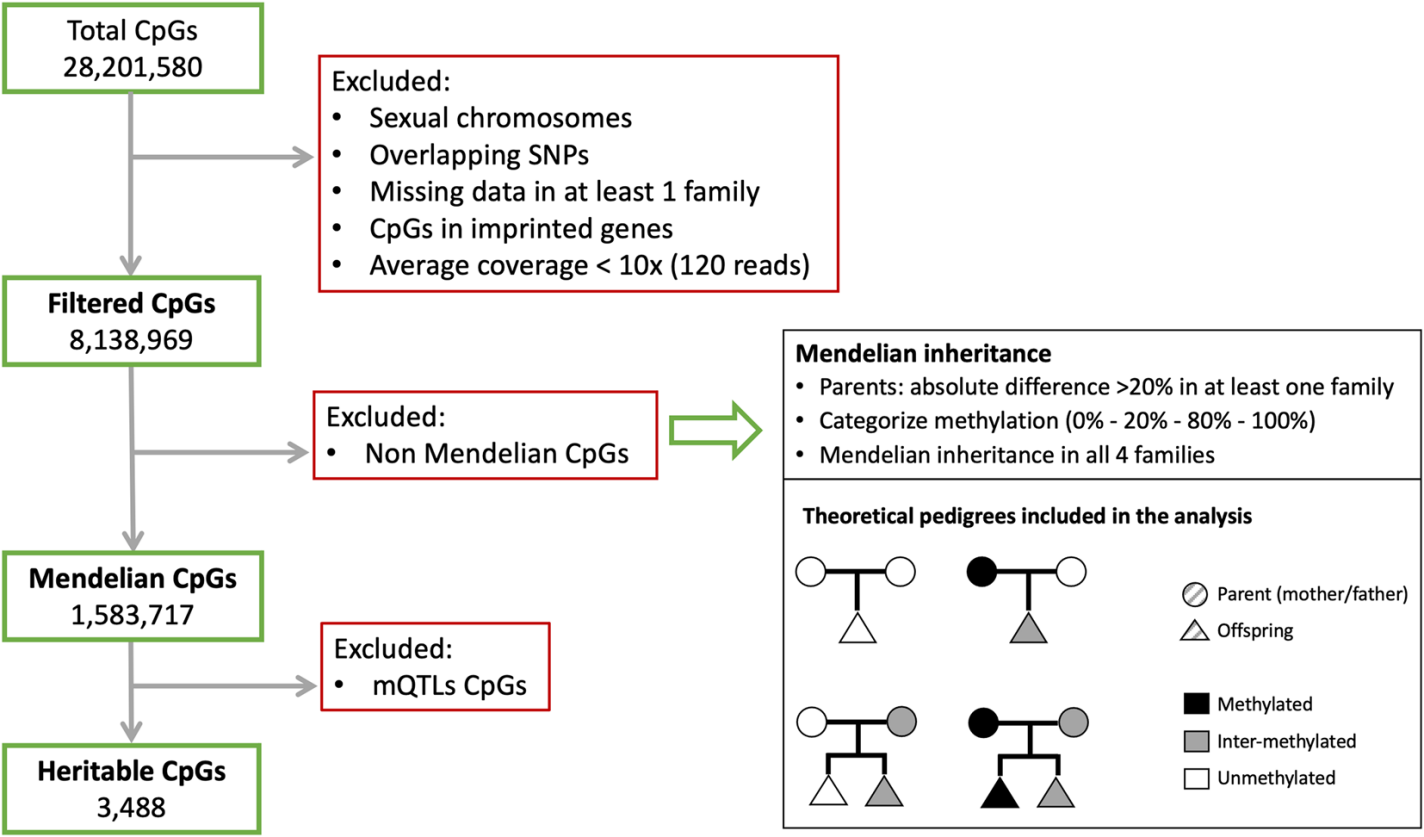
Scheme of the different filters applied to the DNA methylation CpGs to find the three groups analysed. See Abbreviations for definitions. CpGs excluded were 1,198,241 CpGs from chrX and 114,188 CpGs from chrY, 15,707,042 CpGs that overlap with SNPs, 296,703 CpGs with missing data, 156,781 CpGs in imprinted regions and 621,529 CpGs with mean coverage < 10×. The number of filtered CpGs was 8,138,969. From those, 6,555,252 non-Mendelian CpGs were removed to leave 1,583,717 Mendelian CpGs. Finally, 3,488 heritable CpGs were considered after removing 2724 cis mQTLs and 1,577,505 trans mQTLs. On the right, the different accepted Mendelian pedigree schemes are shown.

An outline of the criteria and output for the selection of CpGs considered in the study is shown in Fig. 1. Only CpGs in autosome chromosomes, with a minimum average coverage of 10× (120 reads), with no missing values in at least 3 complete families and that were not SNPs (see “Genotyping data” section) were included in the analysis. Moreover, 156,781 CpGs in imprinted regions were also removed based on a list of 363 common imprinted genes obtained from two sources: 202 genes from Tucci et al.^31^ and 219 genes from gene imprint webpage (https://www.geneimprint.com/). Finally, 8,138,969 CpGs remained for the analysis. We will name these CpGs, “filtered CpGs”. The mean coverage for these CpGs was 13×.

To find methylation loci that had a compatible Mendelian inheritance pattern, but showing intra-family variability, we selected CpGs with an absolute difference in methylation between mother and father greater than 20% in at least one family. Then, we categorised each CpG methylation values in 3 groups: methylated (M) if methylation percentage was equal or greater than 80%; intermediate methylated (I) if it was between 20 and 80% and unmethylated (U) if it was equal or less than 20%. We excluded the CpGs that showed a pattern incompatible with a Mendelian inheritance pattern in at least one family. We identified 1,583,717 CpGs that were variable among parents and had a Mendelian inheritance pattern in the four families and were kept for the analysis. We will name these CpGs, “Mendelian CpGs”.

### Genotyping data

Genotyping was conducted using the Multi-Ethnic Genotyping Array (MEGA) array BeadChip (Illumina, San Diego, CA) which contains 2 million SNPs. We removed SNPs in the array that aligned to multiple loci or to no loci, that were duplicated or with a missing rate greater than 2%. We also removed SNPs with Mendelian errors or far from Hardy Weinberg equilibrium. After this quality control, 1,670,485 SNPs remained for the analysis.

Data was prepared for imputation removing monomorphic or multiallelic SNPs. Only SNPs in autosomal chromosomes were included. Finally, 590,584 SNPs were used for the imputation. Whole genome imputation was performed in the Michigan Imputation Server^32^, using the Haplotype Reference Consortium panel (HRC.r1.1.2016) for CEU population as reference^33^. 39,117,105 SNPs were obtained from the imputation but only 6,166,881 SNPs had an information quality index greater than 0.3.

Once we had the quality imputed SNPs, we excluded those inconsistent with a Mendelian inheritance pattern in the 4 families using plink^34^. The final set contained 6,056,211 SNPs.

### Removing DNA methylation loci explained by genetic inheritance

It was expected that most of the methylation of the 1,583,717 Mendelian CpGs shared an inheritance pattern explainable by genetics. To find out if some of them were not, we searched for associations with SNPs. Using the *MatrixEQTL* package from R^35^ we performed a mQTL analysis by looking for the association of each SNP with each CpG site. To identify mQTLs we used a *p*-value threshold of 5e−15 (0.05/1,583,717 CpGs × 6,056,211 SNPs). We aimed to be very conservative, given that our sample size was very small, and explored both cis and trans associations, to exclude potential indirect effects and test whether some CpGs show a heritable pattern not explained by SNPs. Out of the 1,583,717 Mendelian CpGs, 2,724 (0.2%) were explained by SNPs in *cis* (< 1 Mb), and 1,577,505 more were explained by SNPs in *trans*. The remaining 3488 CpGs that were not associated with any SNP (Additional file 2) will be named “heritable CpGs”.

### Genomic elements and annotations

Genomic elements annotations in genome version hg19 were downloaded from UCSC Table Browser^36^. Gene, exome, and transcription start site (TSS) data were obtained from the *ensGene* table. Gene promoter region was defined as a 2 Kb region upstream the TSS.

CpG island annotations were obtained from the *cpgIslandExt* table. If the distance was zero, the CpG was classified as inside a CpG island. If the distance to a CpG island was greater than zero but less than 2 Kb the CpG was classified as inside a CpG island shore region. If the distance was between 2 and 4 Kb the CpG was classified as inside a CpG island shelf region. Finally, if the distance was greater than 4 Kb the CpG was classified as in open sea.

Chromatin state segmentation for each of nine human cell types were obtained from *wgEncodeBroadHmmGm12878HMM* table. We aggregated these chromatin states as: Promoter (active and weak promoter); Poised Promoter (inactive/poised promoter); Enhancer (strong and weak/poised enhancer); Insulator; Transcription (Transcriptional transition and elongation and weak transcribed); Polycomb repressed; Heterochromatin/low signal/Repetitive/CNV (Heterochromatin/low signal and Repetitive/Copy Number Variation).

Distance to genomic elements was obtained using the *distanceToNearest* function from GenomicRanges Bioconductor package (version 1.48)^37^.

### Flanking regions

**Figure 2:**
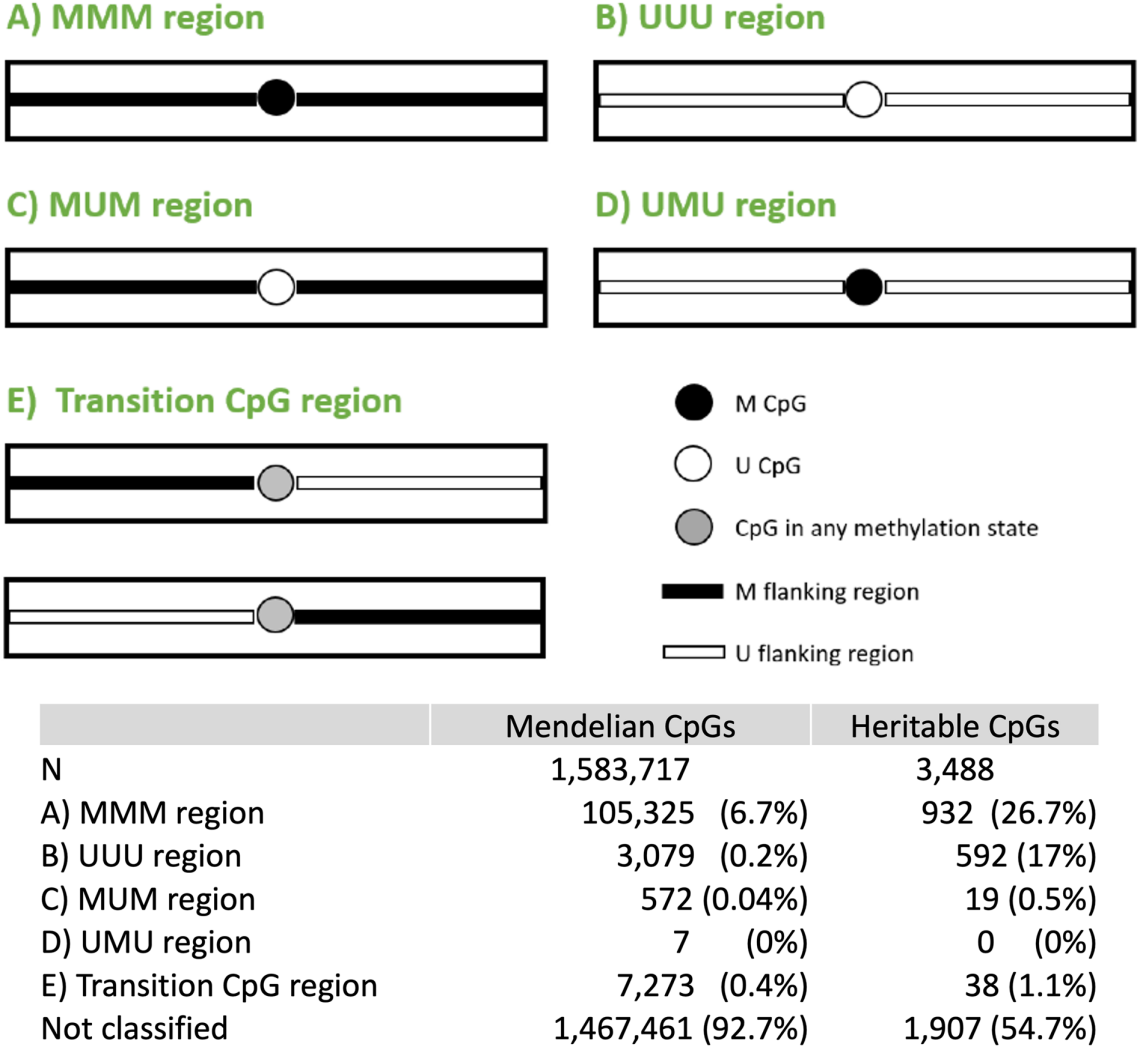
Five different flanking regions scenarios. (**A**) MMM region, defined as a flanking region where more than 80% of the CpGs are methylated and the CpG of interest is also methylated. (**B**) UUU region, defined as a flanking region where more than 80% of the CpGs are unmethylated and the CpG of interest is also unmethylated. (**C**) MUM region, defined as a flanking region where more than 80% of the CpGs are methylated and the CpG of interest is unmethylated. (**D**) UMU region, defined as a flanking region where more than 80% of the CpGs are unmethylated and the CpG of interest is methylated. (**E**) Transition CpG region is denoted by a CpG in any methylation state and flanked by a methylated region and an unmethylated region. The table at the bottom indicates the number of Mendelian and heritable CpGs in each flanking region scenario.

To investigate regional effects, we also analysed the DNA methylation pattern of 1 Kb regions flanking the identified Mendelian and heritable CpGs. For that, we counted the number of methylated (percentage of methylation between 80 and 100%) and unmethylated (percentage of methylation between 0 and 20%) CpGs both upstream and downstream of the CpG of interest (Mendelian or heritable CpG). Based in the proportion of methylated and unmethylated flanking CpGs, we built five scenarios (Fig. 2): (A) MMM region, defined as a flanking region where more than 80% of the CpGs are methylated and the CpG of interest is also methylated. (B) UUU region, defined as a flanking region where more than 80% of the CpGs are unmethylated and the CpG of interest is also unmethylated. (C) MUM region, defined as a flanking region where more than 80% of the CpGs are methylated and the CpG of interest is unmethylated. (D) UMU region, defined as a flanking region where more than 80% of the CpGs are unmethylated and the CpG of interest is methylated. (E) Transition CpG region is denoted by a CpG in any methylation state and flanked by a methylated region and an unmethylated region.

To obtain the GC content of a 1 Kb flanking region of the Mendelian and heritable CpGs we used the *nuc* function from bedtools^38^.

## Results

### Identification of heritable CpGs

This proof-of-principle analysis of 4 trios has identified that from more than 1.58 million CpGs with a DNA methylation pattern variable among parents and compatible with a Mendelian inheritance pattern in the trios, only a relatively small number of them, 3488 (0.2%) were not explained by genetic inheritance assessed by more than 6 million SNPs. Most (99%) of the Mendelian CpGs were explained by SNPs in trans (> 1 Mb).

### Genomic elements distribution

**Figure 3:**
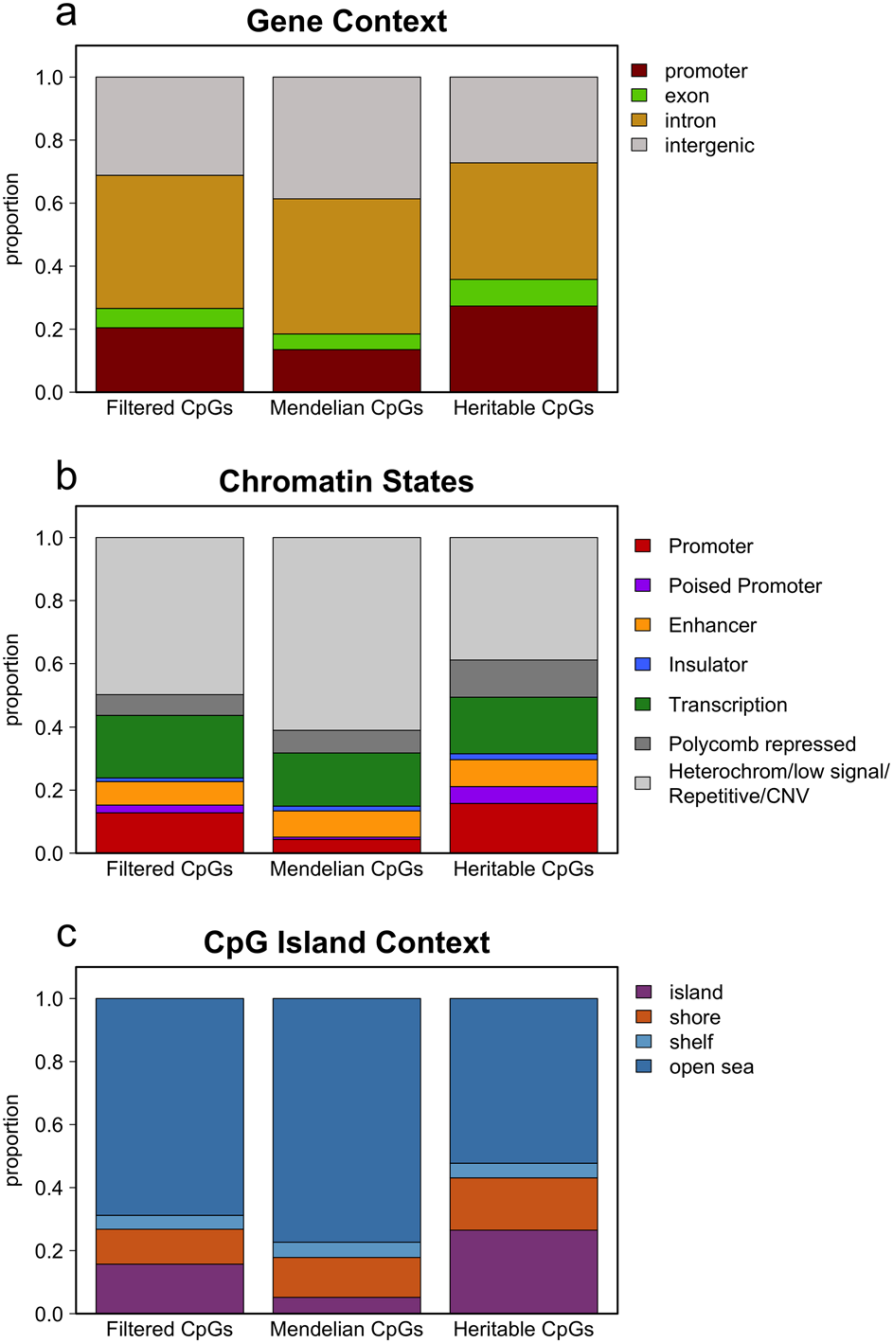
Genomic elements distribution in the three groups of CpGs: filtered, Mendelian and heritable CpGs. (**a**) Proportion of CpGs in the different gene regions. (**b**) Proportion of CpGs in the different chromatin states. (**c**) Proportion of CpGs in the different CpG island regions.

We performed a descriptive analysis of the filtered CpGs, Mendelian CpGs and heritable CpGs to characterise different types of genomic features. Figure 3 shows the distribution of the three groups of CpGs across the different genomic elements. Mendelian CpGs were depleted in promoters (Fig. 3a), promoter chromatin states (Fig. 3b) and CpG islands (Fig. 3c) and enriched in heterochromatin/low signal/repetitive/CNV chromatin states (Fig. 3b). The depletion of Mendelian CpGs in regulatory elements (promoters and CpG islands) has been previously reported by Zaghlool et. al.^39^. Heritable CpGs have a distribution like filtered CpGs in most genomic elements but are more enriched in promoters and CpG islands.

### Distance to genomic elements

We next studied the distance from each CpG to the nearest gene, promoter, TSS region, exon, CpG island, transcription factor binding sites (TFBS) conserved, TFBS cluster, and chromatin state. Summarised data are shown in Additional file 3. If we compare the mean distance between the three groups, we can see that it is lower in heritable CpGs for most of the genomic elements except for the repeats, TFBS conserved and chromatin states.

### DNA methylation profiles

**Figure 4:**
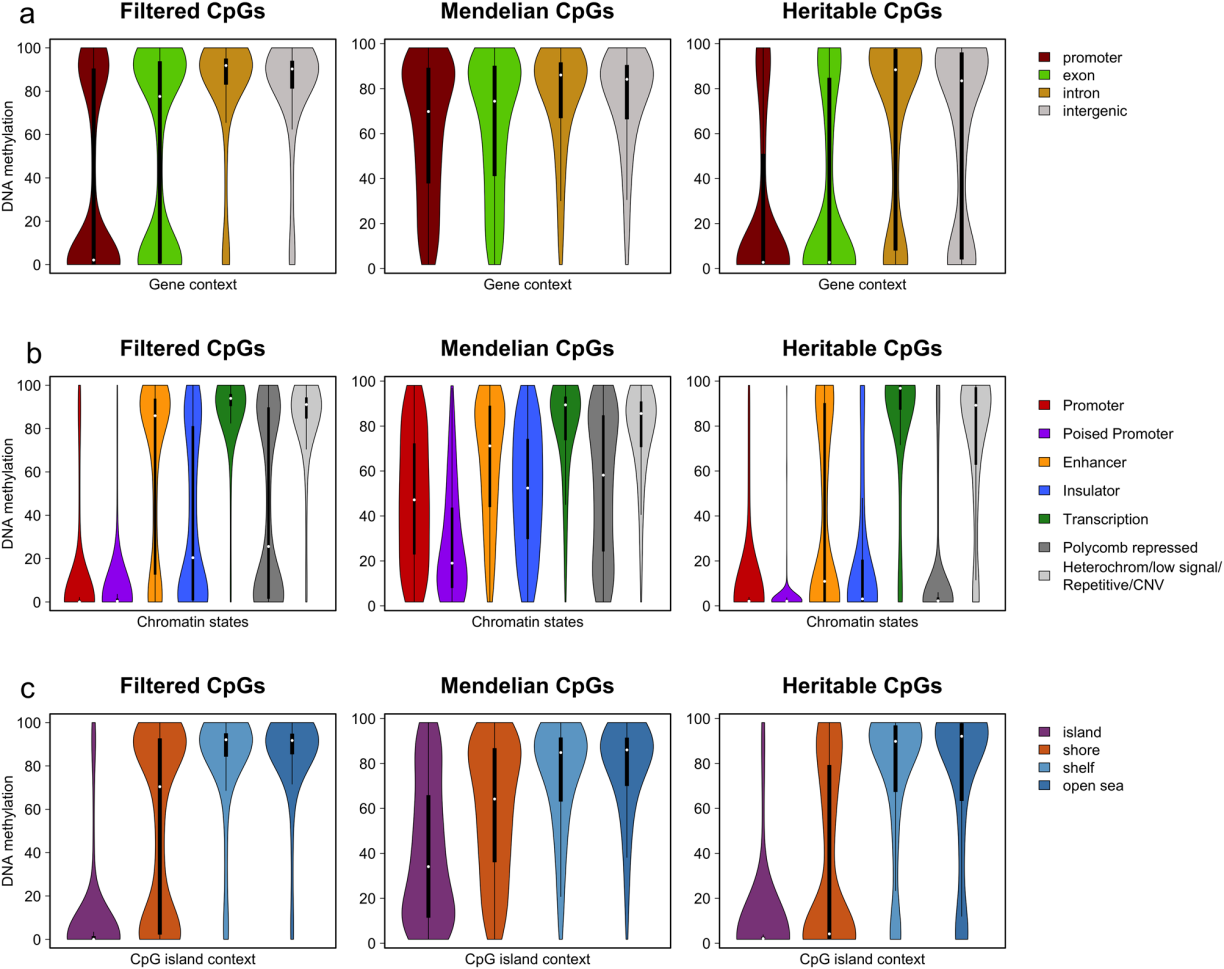
Genomic elements mean methylation distribution in the three groups of CpGs: filtered, Mendelian and heritable CpGs. (**a**) Violin plot of the mean methylation in the different gene regions. (**b**) Violin plot of the mean methylation in the different chromatin states. (**c**) Violin plot of the mean methylation in the different CpG island regions.

We also studied the average methylation distribution of the three groups of CpGs (filtered, Mendelian and heritable CpGs) in relation to their genomic context (Fig. 4).

Regarding gene context, Fig. 4a, Mendelian CpGs, those are CpGs with a Mendelian inheritance pattern that are correlated with SNPs, are highly methylated in all gene regions, even in promoters. Filtered and heritable CpGs are also methylated in intron and intergenic regions but show predominant unmethylation in promoters and exons.

If we look at CpG island context, Fig. 4c, we can see that in CpG islands, filtered and heritable CpGs are unmethylated unlike Mendelian CpGs that tend to have a broad distribution. CpGs located in shores show a bimodal distribution for filtered CpGs. The distribution of DNA methylation values is broad for Mendelian CpGs, while heritable CpGs tend to remain unmethylated. Finally, shelves and open sea regions are methylated in the three groups of CpGs.

**Figure 5:**
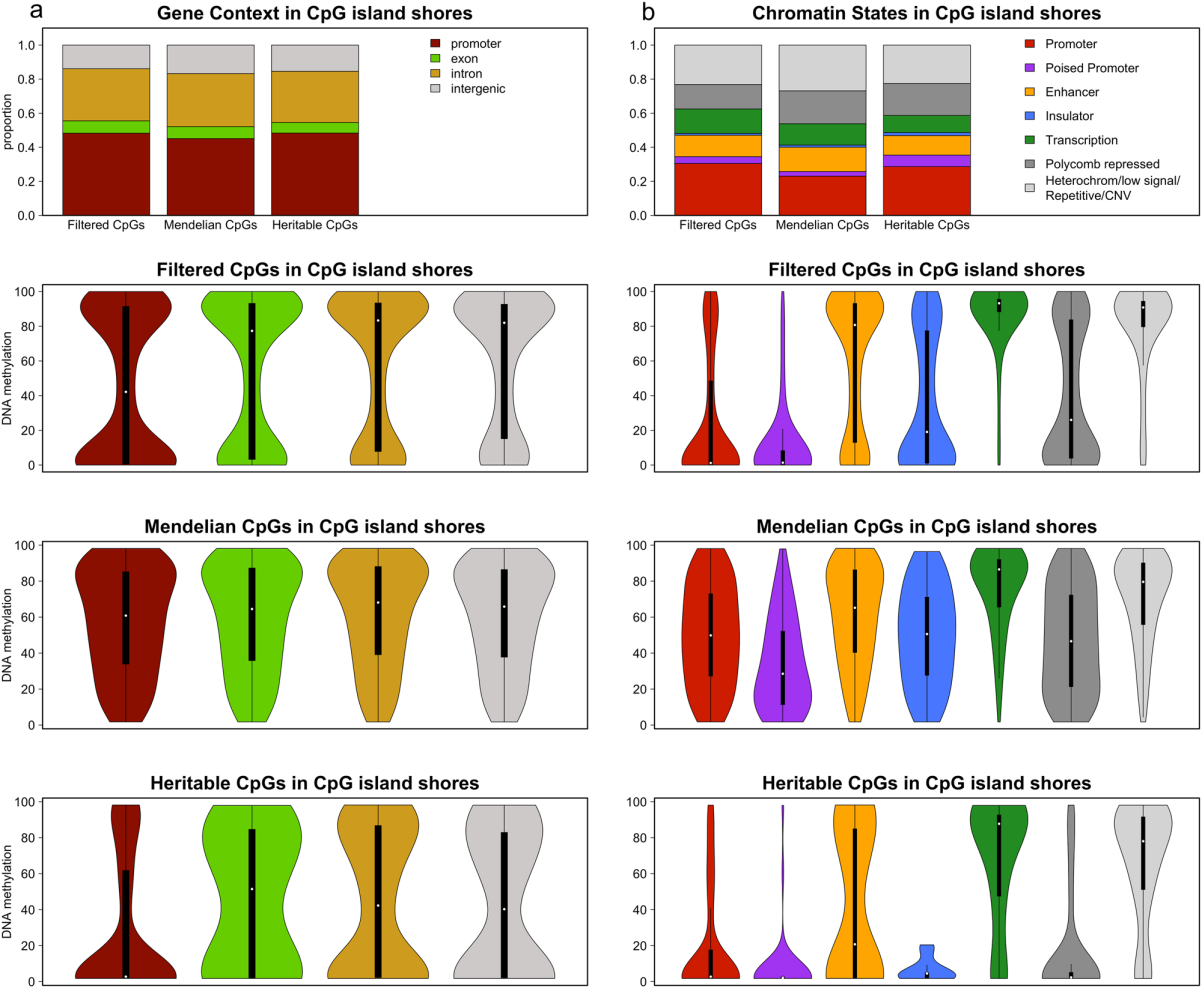
CpGs in CpG island shore regions. Genomic elements distribution and mean methylation profile in the three groups of CpGs: filtered, Mendelian and heritable CpGs. (**a**) Proportion of CpG island shore CpGs and mean methylation profile in the different gene regions. (**b**) Proportion of CpG island shore CpGs and mean methylation profile in the different chromatin states.

Remarkably, past studies have identified CpG island shores as genomic loci with tissue-specific methylation patterns, contrasting with the pervasive methylation of open sea CpGs and the unmethylation of CpG islands^40,41^. This reinforces the interest of the Mendelian and heritable CpGs within CpG island shores as candidate loci with potential phenotypic implications. Figure 5a shows the distribution and methylation profile of CpG island shore CpGs regarding gene context. The three groups of CpGs show similar proportions but very different methylation patterns. While filtered and heritable CpGs show a bimodal distribution in all gene regions, Mendelian CpGs present a broad distribution in all the compartments. Heritable CpGs in promoters and exons tend to be less methylated than filtered or Mendelian CpGs. Figure 5b shows the distribution and methylation profile of shore CpGs regarding chromatin states. The proportion of the different chromatin states is similar in the three groups of CpGs but there are some differences in the methylation distribution. Again, Mendelian CpGs have a broad distribution in most of the chromatin states while filtered and heritable CpGs show a bimodal, a fully methylated or a fully unmethylated pattern. The main difference between filtered and heritable CpGs is that while filtered CpGs tend to be methylated in enhancers, heritable CpGs tend to be unmethylated. These patterns are the same shown in Fig. 4b for the three groups of CpGs, except for enhancers in heritable shore CpGs that are unmethylated in contrast with the global of heritable CpGs showing a broad level of methylation.

### Flanking regions

Figure 2 bottom table displays the distribution of Mendelian and heritable CpGs in each flanking region scenario. The majority of CpGs belong to scenario A (region MMM). Heritable CpGs have a higher proportion of methylated CpGs (26.7%) compared to Mendelian CpGs (6.7%). Scenario B (UUU region) is the second most common scenario, with a higher proportion of heritable CpGs (17%) compared to Mendelian CpGs (0.2%). Scenarios C (MUM region) and D (UMU region), where the methylation pattern of the CpG differs from its flanking region, have very few CpGs with a proportion close to zero in both groups. Finally, despite very few CpGs can be considered a “transition CpG” (scenario E, in which the CpG switches from methylated to unmethylated or vice versa), there is a higher proportion of them in heritable CpGs (1.1%) compared to Mendelian CpGs (0.4%).

Then, we examined the CpGs in scenario E. Additional file 4 describes the distribution of DNA methylation in Mendelian and heritable transition CpGs. Most of the Mendelian transition CpGs show intermediate-high levels of methylation (Additional file 4a) while heritable transition CpGs show a trimodal distribution (Additional file 4b). Finally, the analysis of GC content distribution in the flanking regions showed no major differences between Mendelian and heritable CpGs.

We analysed the similarity of DNA methylation patterns among subjects. Using hierarchical clustering based on Euclidean distance, derived from methylation percentages at each CpG site, we found that children's methylation patterns were more similar to each other than to their parents or other adults. This was observed for both Mendelian and heritable CpGs, as illustrated in Additional file 5a and 5b. This resemblance among children likely stems from our CpG filter criteria, which required diverse DNA methylation patterns in parents. This often resulted in children exhibiting intermediate methylation values. An example of a trimodal methylation pattern is presented in Additional file 5c.

## Discussion

Epigenetic changes are typically thought to be markers of cell differentiation or somatic changes caused by interactions with the environment^16^. However, recent studies have shown that some of these environmentally induced changes can be passed down from one generation to the next, demonstrating their heritability^19,21,42,43^. Though this mechanism has long been known to be relevant in plants^35^, DNA methylation inheritance in mammals has not been studied until recently, other than specific examples^20,21^. Epimutations and imprinting, interconnected mechanisms, are widely recognized for their association with various health conditions. These include cancers, abnormalities in early embryonic development, a range of neurodevelopmental disorders, and diverse congenital anomalies^31,44^.

Genetic variation is an underlying confounding factor in human populations that, independently of any exposure, can lead to stable alterations in epigenetic print^45,46^. This must be considered when looking at epigenetic modifications at a large number of sites in a small set of individuals.

In this study we aimed to develop a strategy to identify epigenetic variations consistent with a Mendelian inheritance pattern, but without an apparent link to genetic transmission as denoted by SNPs. Given the small sample size and sequencing coverage of our study, the results should only be considered a proof of principle that needs validation in a larger study. We have found a small but not negligible number of compatible CpGs, and have performed a genomic characterisation, aiming to discard that these heritable CpGs are just random findings.

Heritable CpGs, showed a distribution through the different genomic regions very similar to the totality of CpGs (filtered CpGs) but with an enrichment in transcription chromatin states. Despite that the methylation profile of heritable CpGs is also very similar to filtered CpGs in most of the genomic elements, heritable CpGs showed more extreme methylation patterns, fully methylated or fully unmethylated. This bimodal methylation status in filtered and heritable CpGs contrasts with other WGBS studies using impurified cell subpopulations, in which intermediate methylated regions are more abundant, showing the need to be careful when drawing conclusions about those methylation results^39,40^. There were two main differences between filtered and heritable CpGs: heritable CpGs tend to be unmethylated in CpG island shores (regions within 2 kb of an island) and tend to have a variable methylation in enhancer chromatin states, while filtered CpGs are methylated in CpG island shores and in enhancer chromatin states. By examining heritable CpGs in CpGs island shores in greater detail, we discovered that they were leading to a decrease in the level of methylation in enhancers.

We have found that heritable CpGs were far from TFBS compared to filtered and Mendelian CpGs. The exclusion of heritable CpGs from TFBS neighbouring regions may imply a negative selection. The association of TFBS changes with variation in DNA methylation profiles contributes to gene regulation through DNA methylation. It is known that once a TFBS becomes unmethylated during differentiation, it remains unmethylated in subsequent stages, suggesting an epigenetic memory of transcription factor binding^41^. This TFBS distribution reveals patterns that support the fact that heritable CpG are unlikely to be cell type specific^42^.

As expected, most of the CpGs that have a Mendelian inheritance pattern are linked to genetic variants^32^. In mammals, DNA methylation in promoter regions with a high density of CpG sites is typically associated with gene silencing, while methylation marks located in coding sequences tend to be associated with the transcription start points. On the other hand, methylation in intergenic regions generally has a lower impact on genomic activity^43^. CpGs with a Mendelian inheritance pattern were found to be depleted in promoter and CpG island regions and enriched in heterochromatin. Additionally, these CpGs showed greater variability in their methylation patterns, including more instances of intermediate methylation in all genomic regions. These changes in methylation at specific nucleotides may indicate the presence of potential binding sites.

Interestingly, some CpGs were found in a DNA methylation transition region and tended to be partially methylated in Mendelian CpGs but either fully methylated or fully unmethylated in heritable CpGs. In this regard, a recent study^47^ has shown that intermediate methylated CpGs present probabilistic patterns of methylation/unmethylation propagation during clonal expansion in vitro. The authors suggest a weighted directionality of methylation inheritance determined by factors as binding of transcription factors.

Finally, we have studied the GC content in 1 Kb flanking region of Mendelian and heritable CpGs. We have seen that a group of heritable CpGs had a flanking region with very high GC content (> 60%) and that these regions are enriched in regulatory elements and CpG island shores and are unmethylated.

It is important to note that our study design has some limitations. Our study was based on a small sample size of 4 family trios, which limits the strength of our results. The provided list of heritable CpGs should be considered preliminary and probably is a subset of a larger number that we don’t have power to detect. Also, some of the identified heritable CpGs may be false positives, because studying a larger sample size could show that they don't really follow a Mendelian inheritance pattern.

The genetic variation largely accounts for the variability in most Mendelian CpGs, with a relatively small subset, numbering 3488, identified as potential candidates for heritable CpGs. This figure likely underrepresents the actual number due to the limited sample size, which restricts the range of possible DNA methylation configurations. Such configurations might coincidentally align with SNPs throughout the genome. Excluding only cis mQTLs could have led to an overestimation of heritable CpGs, given the limited statistical power. However, for this proof-of-principle study, a more conservative approach was favoured, focusing on identifying CpGs whose variations could not be attributed to any SNP.

A second major limitation was the relatively low sequencing coverage. Our goal was a minimum of 10× coverage, constrained by the high sequencing costs at the time of experimentation. Although the sequencing quality was satisfactory, with a median coverage of 11× per sample and a median absolute deviation of 5x, there were challenges. Notably, only 2% of the CpGs analysed had coverage below 5x, but 72% achieved coverage of 10× or higher. The primary concern with low coverage is its impact on the detection of partially methylated CpGs, leading to potential misclassification as fully methylated or unmethylated. To mitigate this, we excluded CpGs with a mean coverage below 10× from our analysis. While this approach reduced the total number of candidate CpGs, it also lessened the likelihood of spurious results.

While WGBS allows us to study both DNA methylation and genetic variation through SNPs, it can misidentify C to T SNPs and C to T bisulphite transformations. Since C to T SNPs are a very common type of SNP in the human population^48^ this can significantly impact our results and make the analysis more challenging, especially considering the limited coverage (10×). To overcome this, we used a genotyping array (Illumina MEGA array) to obtain genetic information for our samples. However, the small sample size of our study resulted in poor quality imputation of the genotyping data and many SNPs were not included in the analysis. It’s also noteworthy that SNP imputation has limitations in detecting rare, family-specific variants, which may have consequential ties to the heritable CpG sites we identified. Furthermore, we must acknowledge the potential influence of other genetic variations—indels, copy number variations (CNVs), microsatellites, inversions, and structural variants—that are not as readily captured by SNP profiling, on DNA methylation patterns^49,50^. Also, polymorphic CpG-dense Alu elements, that are very frequent in the human genome, may explain some of the heritable CpGs. Whole genome sequencing would be a better approach for future studies of this topic.

In this study we analysed neutrophil cells isolated by flow cytometry. This cell purification technique reduces the variation in cell populations in individual samples, making the interpretation of methylation results more reliable compared to other studies^51^, though there are subclasses of these cellular types^52^ that may generate some false positive results if subclasses vary among individuals. Also, it's important to note that neutrophils appear to be primarily influenced by genetics, rather than the environment, based on previous research^51,53,54^. Neutrophils are the most abundant type of leukocyte, but do not divide and have a short half-life of 5 days, which may result in lower requirements for the DNA methylation machinery. On the other hand, other subpopulations with longer lifespans (such as lymphocytes) have been shown to undergo positive and negative selection through different cell divisions. Previous studies have found that granulocytes (which are mostly composed of neutrophils) have a higher concentration of unmethylated regions, while peripheral blood mononuclear cells (PBMCs) are generally more methylated. It is possible that a higher methylation state helps to maintain cellular memory during clonal expansion and regulate the fate of different subpopulations^55,56^.

The optimal validation of our methodology would be to conduct a larger study featuring enhanced coverage and whole-genome sequencing (WGS). In pursuit of preliminary validation, we sought out existing datasets that could corroborate our discoveries. Our search identified a selection of epigenetic investigations on family trios that are complemented by GWAS data^39,46,57^. Predominantly, these studies have used the Illumina Human Methylation 450 K BeadChip assay. This assay includes 473,864 CpG probes in autosomal chromosomes, of which only 35,989 are in our list of Mendelian CpGs and 150 are heritable CpGs. The study by Plongthongkum et al., provided listings of Mendelian CpGs, but they used a different approach, since most of their findings were SNPs, which we have excluded. Unfortunately, we didn’t get an answer when we requested access to these datasets. The Generation Scotland cohort^58^ could potentially serve as a valuable repository for further research. However, it should be noted that accessing this database is contingent upon fulfilling a fee requirement.

Epigenetic mechanisms participate in key cellular processes and specific profiles have been reported to affect susceptibility to certain diseases and response to environmental exposures^16^. Some studies have revealed that heritable, environmentally induced epigenetic alterations can lead to reversible changes in phenotype across generations^13,20,21,42,59^. This transgenerational epigenetic inheritance has been proposed as a potential environment sensing mechanism with an impact in the regulation of biological processes and for the development and progression of sporadic and hereditary diseases^60^. However, transgenerational epigenetic inheritance in animals remains a controversial topic and the underlying mechanisms are poorly understood^24,25,61,62^. Technical challenges and design restraints limit our capacity to demonstrate how non-genetic mechanisms contribute to determining the inheritance of biological features. In this context, the identification of candidate CpGs exhibiting epigenetic inheritance should contribute to open new research avenues to explore their potential biological and evolutionary impact. Additionally, they might provide key resources to investigate the underlying mechanisms. In this study we have carried out a comprehensive methylome analysis in a specific human blood cell type, which reveals a complex landscape of distinct epigenomic features in regulatory sequences and biological information.

In conclusion, this proof of principle study has identified a set of potential heritable CpGs, which constitute less than 1% of all CpGs that follow a Mendelian inheritance pattern. The genomic and epigenomic characterization of these heritable CpGs suggest that they may have functional roles. If these findings are confirmed, the next step would be to study the potential association with diseases and complex traits, to quantify the heritability accounted for by these epigenetic marks, and to identify environmental factors responsible for the DNA methylation changes.

### Supplementary Information

Supplementary Table 1.

Supplementary Table 2.

Supplementary Table 3.

Supplementary Information 4.

Supplementary Information 5.

Supplementary Legends.

## Data Availability

The dataset supporting the conclusions of this article is available in the EGA repository, EGAS00001003018 from the corresponding author on reasonable request.

## Abbreviations

GWAS: Genome-wide association studies
SNP: Single nucleotide polymorphism
mQTL: Methylation quantitative trait loci
WGBS: Whole-genome bisulphite sequencing
MEGA: Multi-Ethnic Genotyping Array
C: Cytosine
T: Thymine
TSS: Transcription start site
TFBS: Transcription factor binding site
PBMC: Peripheral blood mononuclear cell
MMM: Flanking region where more than 80% of the CpGs are methylated and the CpG of interest is also methylated.
MUM: Flanking region where more than 80% of the CpGs are methylated and the CpG of interest is unmethylated.
UMU: Flanking region where more than 80% of the CpGs are unmethylated and the CpG of interest is methylated.
UUU: Flanking region where more than 80% of the CpGs are unmethylated and the CpG of interest is also unmethylated
Filtered CpGs: CpGs in autosome chromosomes, with no missing values in at least 3 complete families and that were not in SNPs or imprinted regions
Mendelian CpGs: Filtered CpGs that had a compatible Mendelian inheritance pattern in the four families, and with an absolute difference in methylation between mother and father greater than 20 in at least one family
Heritable CpGs: Mendelian CpGs that are not associated with any SNP
