# Supplementary figures and images for: Identification of intergenerational epigenetic inheritance by whole genome DNA methylation analysis in trios
Source: Sci Rep. 2023 Dec 2;13:21266. doi: 10.1038/s41598-023-48517-3 (PMC10693549; doi:10.1038/s41598-023-48517-3)

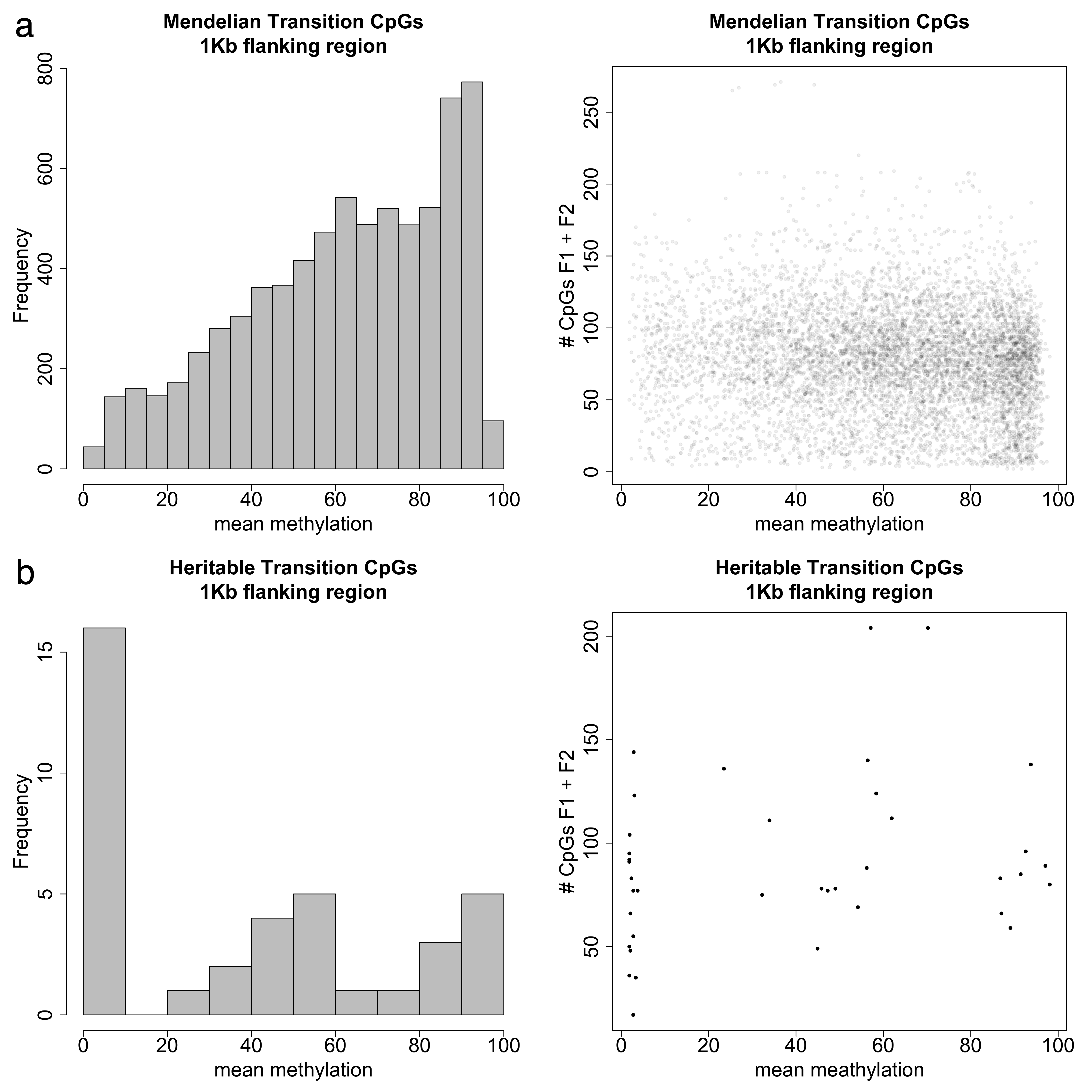

Supplement: Supplementary file 4 — Supplementary Information 4. [file 41598_2023_48517_MOESM4_ESM.tiff]

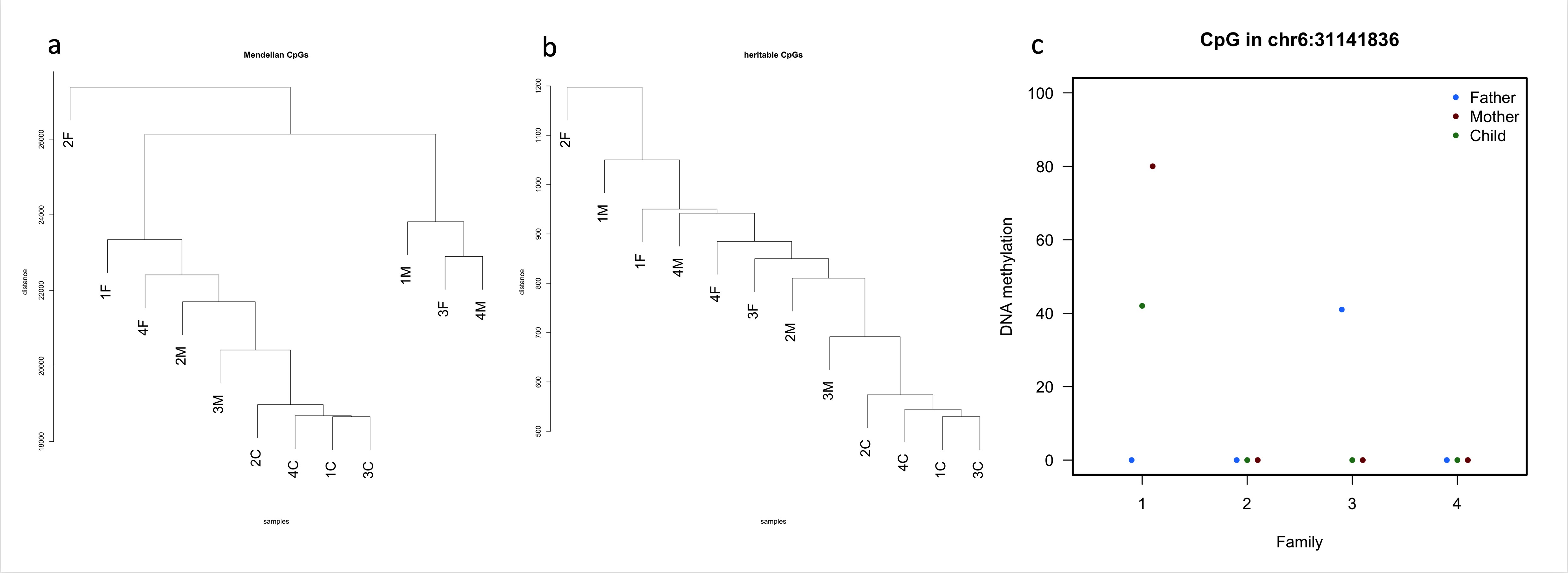

Supplement: Supplementary file 5 — Supplementary Information 5. [file 41598_2023_48517_MOESM5_ESM.tiff]
