# Supplementary material for: Identification of intergenerational epigenetic inheritance by whole genome DNA methylation analysis in trios
Source: Sci Rep. 2023 Dec 2;13:21266. doi: 10.1038/s41598-023-48517-3 (PMC10693549; doi:10.1038/s41598-023-48517-3)
Supplement: Supplementary file 6 — Supplementary Legends. [file 41598_2023_48517_MOESM6_ESM.docx]

**Additional Files Information**

**Additional file 1.xlsx** (15 kb) Samples characteristics and alignment quality control metrics. Qualimap, Picard and FastQC metrics for quality control assessment.

**Additional file 2.xlsx** (336 kb) List of 17,453 heritable CpGs

**Additional file 3.xlsx** (10 kb) Distance to the different genomic elements. Mean, standard deviation (sd) and maximum (max) distance for filtered, Mendelian and heritable CpGs to the different genomic elements.

**Additional file 4.tiff** (1.6 Mb) Mean methylation of the transition CpGs, the CpGs in the flanking region scenario E. **a)** Mendelian CpGs. **b)** Heritable CpGs. First plot shows the mean methylation distribution, and second plot shows the mean methylation versus the number of CpGs in the flanking regions.

**Additional file 5.tiff** (589 kb) Hierarchical cluster dendrogram of the samples, according to Euclidean distance from **a)** Mendelian CpGs and **b)** heritable CpGs. One example of CpG with a trimodal methylation in family 1 and bimodal in family 3 is shown in **c)**.
